# Access of People Living with HIV to Testing Services in Greece: A Challenge for Equity in Care

**DOI:** 10.3390/ijerph192114353

**Published:** 2022-11-02

**Authors:** Kyriakos Souliotis, George Tsiakalakis, Christina Golna, Dimitra Sifaki-Pistolla, Nikos Dedes, Marios Lazanas

**Affiliations:** 1 Faculty of Social and Education Sciences, University of Peloponnese, 20100 Corinth, Greece; 2 Health Policy Institute, 15123 Athens, Greece; 3 Greek Association of People Living with HIV Positive Voice, 10554 Athens, Greece; 4 Internal Medicine and Infectious Diseases Department of IASO General Clinic, Hellenic Society for the Study and Control of AIDS (EEMAA), 15123 Athens, Greece

**Keywords:** HIV, HIV testing, lymphocyte tests, genotype resistance, accessibly, continuity

## Abstract

(1) Background: Access to laboratory testing services for HIV in Greece is persistently challenged and this impacts both the continuum of care and, potentially, equity in access. (2) Methods: A cross-sectional study with two parts (first part: HIV-positive people/PLWHIV; second part: HIV clinicians) was conducted in Greece to quantify challenges regarding access to laboratory testing for HIV. Data were collected through online surveys, during a one-month period, between 2019 and 2020. The total sample consisted of 153 PLWHIV and 26 HIV clinicians. (3) Results: Access to viral load testing varied significantly according to place of residence (*p* = 0.029) and year of diagnosis (*p* = 0.054). Patients diagnosed after 2015 reported worse access to viral load testing (72.7% vs. 85.9%). Over one third of respondents perceived viral load tests as being not at all accessible (11.4%) or somewhat accessible, only after facing multiple systemic obstacles (24.2%). Equally, most of HIV clinicians reported barriers or no access to baseline viral load testing (80%) and baseline genotype resistance tests (96%). (4) Conclusions: Access of people diagnosed with HIV to CD4 lymphocyte tests and genotype resistance screening is significantly challenged in Greece, especially after 2015. Addressing this challenge is critical in removing access barriers and achieving the UNAIDS 95-95-95 HIV elimination goals.

## 1. Introduction

Scientific advances in HIV treatment over the last 25 years have transformed the condition into a chronic one and provided patients with a satisfactory quality of life [1]. The Joint United Nations Programme on HIV/AIDS (UNAIDS) in its latest HIV Strategy [2] calls for bold elimination goals that include at least 95% of people living with HIV (PLWHIV) knowing their status, 95% of those being linked to appropriate care and receiving antiretroviral treatment (ART) and 95% of the latter being virally suppressed. To achieve these bold goals the strategy “keeps PLWHIV at its center to ensure that they benefit from optimal standards in service planning and delivery, to remove social and structural barriers that prevent them from accessing HIV services, to empower communities to lead the way, to strengthen and adapt systems so they work for the people who are most acutely affected by inequalities, and to fully mobilize the resources needed to end AIDS” [2].

Such priorities are clearly aimed to address inequalities in access to care that may be formed or affected by disparities and inefficiencies in care delivery as well as health services and community stigma [3]. Irrespective of source of such inequalities, their impact on equity in access is undeniable [3,4]. Failure to ensure equity in access to HIV diagnosis and care is doubly critical in terms of both individual and public health outcomes, as late diagnosis and not achieving swift viral suppression exposes the person living with HIV to disease progression and complications and the community to potentially additional infections [5].

In Greece, HIV management has traditionally been considered a well-managed area of health care and a paradigm for efficient use of healthcare resources [6]. Systematic epidemiological surveillance by the National Public Health Organization (EODY), a comprehensive registry for PLWHIV under care, which records any treatment regimens dispensed [7], coupled with physician’s freedom in treatment choice according to individualized conditions, and patient management by Infection Disease Units (IDUs) and HIV Clinics within the NHS, have contributed to this efficient disease management. Nonetheless, it seems that there remain significant challenges with access to HIV diagnostic tests, baseline screening, and laboratory monitoring services [7]. Such services have until very recently been reserved for public laboratories only, with reagents tendered centrally through the National Retroviral Reference Center (EKAR), whereas access to reimbursed testing services in the private sector has only recently been introduced, with significant challenges remaining as regards the safeguarding of anonymity and confidentiality of data of PLWHIV. Meanwhile, delays in central tenders by EKAR have repeatedly challenged access to services even within the NHS.

As a result, it has been increasingly difficult to follow both Greek [8,9] and European [10] guidelines in clinical practice. Both recommend that CD4 lymphocyte and viral load screening be performed at least twice a year. Genotype resistance testing is recommended before starting the first ART regimen, ideally at the time of diagnosis or in case of a superinfection [8,9,10]. Delays in performing such testing may impact on rapid initiation of treatment or treatment optimization and as such they generate inequities for PLWHIV, jeopardize universal access to laboratory tests, and may have major implications for epidemiological surveillance and, ultimately, public health [9,10]. Lastly, it should be noted that there are Greek studies highlighting the increased unmet health needs of HIV positive people, especially during and after the era of the financial crisis [7]. These needs had to be managed with decreasing available funds, which might have impacted the access to and quality of offered services [11].

This study reports on challenges related to accessing laboratory testing services for HIV in Greece (CD4 lymphocytes, viral load, genotype resistance) to quantify its potential impact on the continuum of care that affects both individual and public health outcomes, as well as equity in access to health care services overall.

## 2. Materials and Methods

### 2.1. Study Design

The present research is a cross-sectional study with two parts. The first part was addressed to PLWHIV and the second part was addressed to HIV clinicians employed in Infectious Disease Units of the National Health System (NHS) across the country. Data were collected through online surveys, one for each part of the survey, during a one-month period, between 10 December 2019, and 10 January 2020. Geographical regions are reported using three categories (i.e., Attica, Thessaloniki and rest of Greece). Attica is the capital region of Greece, representing the biggest, highest populated urban city. Thessaloniki is the second biggest city of medium density, while rest of Greece is comprised of semi-urban and rural cities and villages. The categorization in three major regions is performed in line with the concentration of PLWHIV in treatment centers for care, with the vast majority in Athens [12].

### 2.2. Study Population and Procedures

Study sample consisted of 153 PLWHIV, who were members of the HIV Patient Organization “Positive Voice”, as well as 26 HIV clinicians employed in the 20 Infectious Disease Units & HIV Outpatient Clinics of the National Health System (NHS), who were members of the Hellenic Society for the Study and Control of AIDS (EEMAA).

Purposive sampling (non-probabilistic approach) was used to select study sample, as both PLWHIV and HIV clinicians are considered special groups to identify for the needs of a random or other probabilistic sampling. An email was sent separately to the Boards of Directors of both Positive Voice and EEMAA along with the study’s protocol and instrument for approval. Both agreed to participate in the study and sent a participation invitation, an informed consent form, information regarding the study and a link to each respective questionnaire to its members. All recipients had the same chance of participating in the study.

According to the National Organization for Public Health (EODY) [13], in 2021, there were 526 new HIV diagnoses and approximately 17,113 people diagnosed and living with the condition in Greece, of which 10,618 were on Anti Retrovirtal Treatment (ART) on 31 December 2019 [14].

### 2.3. Research Tool

The questionnaire addressed to patients included questions on the socio-demographic characteristics of participants (gender, age, educational level, and place of residence), on the disease (time of diagnosis, time on treatment, treatment optimization) and questions regarding the accessibility and availability of laboratory testing during diagnosis, baseline screening and regular monitoring. Respondents were also asked if they were aware of their current HIV status (viral load detectable or not).

The questionnaire addressed to HIV physicians included questions on the socio-demographic characteristics of participants (gender, age, years of experience, and place of employment) and questions regarding the accessibility and availability of laboratory tests for their patients -PLWHIV.

### 2.4. Pilot Study

We conducted a pilot study to evaluate the effectiveness of the survey questionnaire in answering the research question amongst the first group (PLWHIV). The questionnaire was pre-tested with two representatives of “Positive Voice” to ensure validity, clarity in language, and ability to be clearly understood by respondents. Feedback from the pilot study was incorporated into the final questionnaire. Responses provided during the pilot were excluded from the final sample and analysis.

### 2.5. Statistical Analysis

All *p*-values reported are two-tailed. Statistical significance was set at 0.05 and analyses were conducted using SPSS statistical software (IBM, New York, USA, version 24.0). Chi-square and Fisher’s exact tests were used to compare the observed proportions.

## 3. Results

Table 1 presents sample sociodemographic characteristics both for PLWHIV (n = 154) and HIV specialists (n = 26). PLWHIV who participated in the survey were diagnosed on average 9.21 years ago (SD = 6.63, range from 0 to 34 years) and had been receiving antiretroviral treatment for an average of 7.64 (SD = 5.95) years.

Most participants stated that baseline CD4 testing was quite (39.5%) or very (47.4%) accessible; however, over 1 in 10 considered it either not accessible at all (4.6%) or accessible only after overcoming a series of systemic obstacles (8.6%). 73.9% stated that they underwent a viral load test (HIV RNA) within 2 weeks of diagnosis, while 17.6% did not.

Access to viral load testing varied significantly according to place of residence (×2 (1140) = 4778 *p* = 0.029) and year of diagnosis (×2 (1140) = 3712, *p* = 0.054). Patients diagnosed after 2015 stated worse access to viral load testing compared to those diagnosed before 2015 (72.7% vs. 85.9%, respectively). Regarding place of residence, patients who lived outside of Athens (69.8%) were also less able to undergo viral load testing compared to residents of Athens (85.6%) (Table 2).

Regarding their most recent monitoring tests, 30.7% of respondents reported not receiving their latest CD4 lymphocyte test results and 50% reported not receiving their most recent viral load (HIV RNA) test results within 2 weeks. There was no statistically significant difference between patients’ place of residence (CD4: ×2 (1.142) = 0.663, *p* = 0.416 and Viral load: ×2 (1.138) = 1.370, *p* = 0.242, respectively) or the year of diagnosis (CD4: ×2 (1140) = 0.149, *p* = 0.699 and Viral load: ×2 (1136) = 1.025, *p* = 0.311) and acquiring or not results of monitoring tests within 2 weeks (Table 3).

In addition, 35.3% of the PLWHIV sample reported visiting a private laboratory for a viral load test in the last 12 months. There was no statistical difference between this choice and place of residence (×2 (1.152) = 0.421, *p* = 0.517) or year of diagnosis (×2 (1.150) = 1.487, *p* = 0.223). At the time of the study, over one-third of respondents perceived viral load tests as being not at all accessible (11.4%) or somewhat accessible, only after facing multiple systemic obstacles (24.2%) (Table 3).

Equally, most of HIV clinicians in our sample (88.4%) considered that baseline CD4 lymphocyte screening is very or quite accessible (Table 4). On the contrary, the majority reported barriers or no access to baseline viral load testing (80%) and baseline genotype resistance tests (96%). 48% reported genotype testing as being not accessible at all. They reported delays of over 2 weeks in acquiring test results in all screening tests, with significant delays in viral load and genotypic resistance screening. Delays were primarily attributed to lack of reagents (65% viral load, 63.6% genotype resistance) and resources (15% viral load, 18.2% genotype resistance).

During monitoring, HIV specialists reported the lowest accessibility for viral load and genotype resistance testing in treatment naive patients. Treatment-experienced patients also suffered limited accessibility to viral load and genotype resistance testing for monitoring purposes as well as delays. These delays were considered particularly important by HIV clinicians both in treatment naïve (62.5% viral load testing and 81.8% genotypic resistance testing) and treatment-experienced patients (62.5% and 82.6%, respectively) and were mainly attributed to lack of reagents (61.9% and 68.2%, respectively, both treatment naïve and experienced patients) (Table 4).

Finally, more than 1 in 2 HIV clinicians reported having 0 to only 15% records of genotypic resistance data for PLWHIV diagnosed after 2015 (M = 15%, interquartile range = 30%).

## 4. Discussion

The aim of this study was to investigate barriers faced by PLWHIV in Greece with accessing laboratory tests for CD-4 lymphocytes, viral load, and genotype resistance. According to our findings, 17.6% of the PLWHIV sample did not undergo viral load testing (HIV RNA) within 2 weeks of their diagnosis, a finding that is statistically significantly correlated with year of diagnosis and place of residence. In addition, there is a marginally significant correlation between place of residence and time since the last HIV RNA viral load test, whereas 1 in 3 respondents reported perceiving them as not at all (11.4%) or somewhat (24.2%) accessible or available, after first having to overcome a series of systemic obstacles. Corresponding findings of the survey amongst HIV clinicians record a worse picture for access to laboratory testing, especially when it comes to viral load and genotypic resistance testing, compared to findings in the PLWHIV sample. This discrepancy could be attributed to the characteristics of the PLWHIV sample, all of whom are members of a strong Patient Association and exposed to or with access to patient advocacy.

Almost all study participants (99.3%) stated that they were receiving ART and 82.20% reported being aware of their viral load status. The latter indicator is higher than that reported previously. In the official European Center for Disease Control (ECDC) report of 2018, Greece did not report any data on the percentage of patients with undetectable viral load [15]. Further, in a study by Vourli et al. (2018) [16] of the cascade of HIV care in 11 European countries, it was estimated that 73% of PLWHIV in these countries had successfully suppressed their viral load, with a significant variation between Greece (58.7%) and Denmark (86%). It was specifically mentioned that as early as 2016, Greece was faced with bureaucratic and financial challenges that affected access to laboratory testing. As a result, the country recorded the lowest rates, although the real proportion of people with undetectable viral load may be expected to be higher.

Our findings are in line with previously reported challenges and barriers in access to HIV-related health care services in other developed [17] or developing countries [18]. Although data on PLWHIV and health care accessibility in Greece, are lacking, there are some insights on such barriers for patients with chronic diseases. For instance, geographical disparities in access to cancer care have been reported from other studies conducted in Greek settings. Particularly, in Athanasakis et al., (2012) [6], cancer patients had to travel extensively to access appropriate care, if they resided outside of Athens, thus creating a two-tier cancer patient matrix. Such system shortcomings have been confirmed elsewhere [19,20,21,22,23]. In the HOPE studies [19,20,21], patients with rheumatoid arthritis [19], cancer [20], multiple sclerosis [21] and drug users with hepatitis C [22] reported critical barriers in accessing medicines, physical distance from providers and long waiting times for a medical appointment [7]. Similar barriers in access to health care were also reported for patients with beta-thalassemia in Greece, especially for those residing away from transfusion centers and have lower income [24]. Therefore, any challenges faced by people with HIV across their patient pathway, and in particular during screening for CD4 lymphocytes, viral load, and genotype resistance, should be viewed as part of wider reforms required in the NHS to ensure operational efficiency [25] and patient accessibility.

Yet, unlike these other chronic conditions, HIV is an infectious disease, where diagnosis, linkage to the healthcare system, early access to antiretroviral therapy, monitoring and screening according to the guidelines, constitutes a necessary condition for not only effectively managing the condition for the person living with it but also addressing a persistent challenge for public health [26]. Studies have demonstrated benefits of antiretroviral therapy initiation as soon as a person is diagnosed [27]; however, it should be accompanied by systematic and standardized laboratory testing confirmation [28]. It is, therefore, even more critical to remove all barriers in access to services across the pathway, if to ensure optimal individual and public health outcomes. Inability to access critical services, including laboratory testing, even by some PLWHIV, may still negatively impact public health outcomes and contribute to the health system not achieving the UNAIDS elimination goals [2].

### Limitations

This study was conducted via email, therefore, PLWHIV with no registered email with Positive Voice did not have access to it. This may have introduced sampling bias, as it is likely that extremely vulnerable or marginalized PLWHIV with no access to the internet or no computer skills may not have been able to participate. Additionally, the purposive sampling approach may have inserted external validity biases; however, the authors believe that the current finding strongly represent the reality PLWHIV and HIV professionals are facing in Greece. All findings depict current trends.

## 5. Conclusions

This is the first study to quantify impact of barriers to access to laboratory testing for HIV amongst PLWHIV in Greece. Despite HIV management being a paradigm of efficient use of resources over the past decade [29], challenges with access to laboratory testing particularly for CD4 lymphocyte tests, viral load, and genotype resistance screening may critically undermine the ability of the health care system to achieve HIV elimination goals and impact on equity in access to care indicators for PLWHIV across the country.

## Figures and Tables

**Table 1:** Socio-demographic characteristics of the sample.

PLWHIV	
**Gender**	**Ν (%)**
Man	150 (98.0%)
Woman	3 (2.0%)
**Age**	
18–24	4 (2.6%)
25–34	31 (20.3%)
35–44	54 (35.3%)
45–54	49 (32.0%)
55–64	13 (8.5%)
65+	2 (1.3%)
**Educational level**	
Compulsory education	5 (3.3%)
Secondary education	36 (23.5%)
Tertiary education	75 (49.0%)
Master’s degree/PhD	37 (24.2%)
**Place of residence**	
Attica	109 (71.2%)
Thessaloniki	24 (15.7%)
Rest of Greece	20 (13.1%)
**HIV clinicians**	
**Gender**	
Man	14 (53.8%)
Woman	12 (46.2%)
**Age**	
25–34	2 (7.7%)
35–44	3 (11.5%)
45–54	11 (42.3%)
55–64	10 (38.5%)
**Years of specialization**	
0–5	1 (3.8%)
5–10	1 (3.8%)
10–15	10 (38.5%)
15+	14 (53.8%)
**Place of employment**	
Attica	23 (88.5%)
Thessaloniki	1 (3.8%)
Rest of Greece	2 (7.7%)

**Table 2:** Baseline Screening for CD4 lymphocytes and viral load (HIV RNA), within 2 weeks of diagnosis.

Within 2 weeks of your diagnosis, did you undergo a CD4 lymphocyte test?	
No	15 (9.8%)
Yes	125 (81.7%)
I don’t remember	13 (8.5%)
**If yes, did you receive the results of your test within 2 weeks?**	
No	6 (4.2%)
Yes	130 (90.3%)
I don’t remember	8 (5.6%)
**How accessible/available was this test to you (2 weeks after diagnosis)?**	
Not at all	7 (4.6%)
Somewhat (after facing many systemic obstacles)	13 (8.6%)
Fairly	60 (39.5%)
Very much	72 (47.4%)
**Within 2 weeks of your diagnosis, did you undergo a viral load test (HIV RNA)?**	
No	27 (17.6%)
Yes	113 (73.9%)
I don’t remember	13 (8.5%)
**If yes, did you receive the results of your test within 2 weeks?**	
No	26 (17.9%)
Yes	106 (73.1%)
I don’t remember	13 (9.0%)
**How accessible/available was this test to you (2 weeks after diagnosis)?**	
Not at all	24 (15.8%)
Somewhat (after facing many systemic obstacles)	16 (10.5%)
Fairly	47 (30.9%)
Very much	65 (42.8%)

**Table 3:** Patient responses on most recent testing results.

Did you receive the results of your CD4 lymphocyte test within 2 weeks?	
No	46 (30.7%)
Yes	96 (64.0%)
I don’t remember	8 (5.3%)
**Did you receive the results of your HIV RNA test within 2 weeks?**	
No	74 (50.0%)
Yes	64 (43.2%)
I don’t remember	10 (6.8%)
**Did you undergo a viral load test in a private lab within the last 12 months?**	
No	98 (61.4)
Yes	54 (35.3)
I don’t remember	1 (0.7)
**At this time, how accessible/available would you describe viral load testing to you?**	
Not at all	17 (11.4%)
Somewhat (after facing many systemic obstacles)	36 (24.2%)
Fairly	47 (31.5%)
Very much	49 (32.9%)

**Table 4:** HIV Specialists’ responses on testing accessibility.

	Baseline Screening before Starting Treatment	Monitoring in Treatment Naïve Patients	Monitoring in Treatment Experienced Patients
**Accessibility of CD4 lymphocyte screening tests**			
Very much	16 (61.5%)		
Fairly	7 (26.9%)		
Somewhat (after facing many systemic obstacles)	2 (7.6%)		
Not at all	1 (4.0%)		
**Accessibility of viral load tests**			
Very much	3 (12.0%)	4 (16.7%)	4 (16%)
Fairly	2 (8.0%)	0	0
Somewhat (after facing many systemic obstacles)	18 (72.0%)	15 (62.5%)	14 (56%)
Not at all	2 (8.0%)	5 (20.8%)	7 (28%)
**Accessibility of genotype resistance tests**			
Very much	1 (4.0%)	2 (8.3%)	2 (8%)
Fairly	0	0	0
Somewhat (after facing many systemic obstacles)	12 (48.0%)	9 (37.5%)	11 (44%)
Not at all	12 (48.0%)	13 (54.2%)	12 (48%)
**Time span of receiving viral load results**			
0–24 h	0	0	0
25–72 h	3 (12.5%)	2 (8.3%)	2 (8.3%)
7–14 days	4 (16.7%)	6 (25%)	6 (25%)
More than two weeks	17 (16.8%)	16 (66.7%)	16 (66.7%)
**Time span of receiving genotypic resistance test results**			
0–24 h	0	0	0
25–72 h	0	0	0
7–14 days	1 (4.3%)	1 (4.8%)	1 (4.5%)
More than two weeks	22 (95.7)	20 (95.2%)	21 (95.5%)

## Data Availability

The dataset used and analyzed during the current study is available from the corresponding author on reasonable request.
